# Supplementary material for: Prevention of necrotizing enterocolitis with probiotics: a systematic review and meta-analysis
Source: PeerJ. 2016 Oct 5;4:e2429. doi: 10.7717/peerj.2429 (PMC5068355; doi:10.7717/peerj.2429)

**Appendix A: Examples of Detailed Search Strategies**

**Medline and Central (via Ovid)**

1 neonat*.mp. (9409)

2 exp Infant, Newborn/ or infant*.mp. (33861)

3 newborn*.mp. (14783)

4 1 or 2 or 3 (38396)

5 exp Probiotics/ or probiotic*.mp. (1847)

6 exp Lactobacillus delbrueckii/ or lactobacillus.mp. or exp Lactobacillus plantarum/ or exp Lactobacillus fermentum/ or exp Lactobacillus leichmannii/ or exp Lactobacillus casei/ or exp Lactobacillus helveticus/ or exp Lactobacillus rhamnosus/ or exp Lactobacillus acidophilus/ or exp Lactobacillus/ or exp Lactobacillus reuteri/ or exp Lactobacillus brevis/ (1705)

7 bifidobacterium.mp. or exp Bifidobacterium/ (737)

8 exp Saccharomyces cerevisiae/ or exp Saccharomyces/ or saccharomyces.mp. (209)

9 5 or 6 or 7 or 8 (2792)

10 4 and 9 (704)

11 limit 10 to controlled clinical trial (22)

12 limit 10 to randomized controlled trial (400)

13 11 or 12 (418)

14 limit 13 to yr="2013 -Current" (32)

15 necrotizing enterocolitis.mp. or exp Enterocolitis, Necrotizing/ (430)

16 necrotising enterocolitis.mp. (60)

17 15 or 16 (472)

18 limit 17 to controlled clinical trial (28)

19 limit 17 to randomized controlled trial (233)

20 18 or 19 (261)

21 limit 20 to yr="2013 -Current" (19)

22 14 or 21 (48)

**Embase (via Ovid)**

1 exp probiotic agent/ or probiotic*.mp. (21366)

2 exp Lactobacillus reuteri/ or exp Lactobacillus helveticus/ or lactobacillus.mp. or exp Lactobacillus paracasei/ or exp Lactobacillus rhamnosus/ or exp Lactobacillus bifidus/ or exp Lactobacillus acidophilus/ or exp Lactobacillus/ or exp Lactobacillus casei extract/ or exp Lactobacillus casei/ (33651)

3 bifidobacterium.mp. or exp Bifidobacterium breve extract/ or exp Bifidobacterium longum/ or exp Bifidobacterium/ or exp Bifidobacterium breve/ or exp Bifidobacterium longum infantis/ (8405)

4 exp Saccharomyces cerevisiae/ or exp Saccharomyces/ or exp Saccharomyces boulardii/ or saccharomyces.mp. (97029)

5 1 or 2 or 3 or 4 (142940)

6 exp prematurity/ or neonate*.mp. (161442)

7 newborn/ (505429)

8 6 or 7 (569827)

9 5 and 8 (1646)

10 limit 9 to randomized controlled trial (140)

11 limit 9 to controlled clinical trial (100)

12 10 or 11 (160)

13 limit 12 to yr="2013 -Current" (35)

14 necrotising enterocolitis.mp. (952)

15 necrotizing enterocolitis.mp. or exp necrotizing enterocolitis/ (8591)

16 14 or 15 (9117)

17 limit 16 to randomized controlled trial (460)

18 limit 16 to controlled clinical trial (355)

19 17 or 18 (538)

20 limit 19 to yr="2013 -Current" (91)

21 13 or 20 (115)

**Conference Meetings**

| Society of Pediatric Research |  |  | |  |  |
| --- | --- | --- | --- | --- | --- |
| **Meeting** | **Date** | | **Citations** | **Excluded** | **Included** |
| Pediatric Academic Societies (PAS)/Asian Society for Pediatric Research | 2014 | | 6 | 6 | 0 |
|  | 2015 | | 40 | 38 | 2  (titles only) |
|  | 2016 | | 17 | 16 | 1  (ProPrems data) |
| PAS Late-Breaking abstracts | 2014 | | 0 | 0 | 0 |
| The Eastern Society for Pediatric Research | 2014 | | 6 | 6 | 0 |
|  | 2015 | | 4 |  |  |
|  | 2016 | | 27 | 27 | 0 |
| The Midwest Society for Pediatric Research | 2015 | | 3 | 3 | 0 |
| The Southern Society for Pediatric Research | 2016 | | 3 | 3 | 0 |
| The Western Society for Pediatric Research | Not searchable online | |  |  |  |

| European Society of Pediatric Research |  |  |  |  |
| --- | --- | --- | --- | --- |
| **Meeting** | **Date** | **Citations** | **Excluded** | **Included** |
| European Academy of Paediatric Societies | 2014 | 2 | 2 | 0 |
|  | 2015 | 7 | 7 | 0 |

| Clinical trial registries | | | | |
| --- | --- | --- | --- | --- |
| **Registry Name** | **Date** | **Citations** | **Excluded** | **Included** |
| National Institute of Health (clinical trials.gov) | Jan 2015  June 2016 | 24 | 24 | 0 |
| WHO International Clinical Trials Registry Platform | Jan 2015  June 2016 | 11 | 11 | 0 |

**Appendix B: List of data extracted from included trials**

**Trial Characteristics**

- number of participating centres and countries
- inclusion criteria
- exclusion criteria
- number of patients eligible/randomized
- treatment & follow-up duration
- treatment arms in the trials
- species of probiotic used and if multiple species or not
- timing of probiotic initiation and duration of treatment
- ethics and consent
- funding source

**Participant Characteristics**

- number of patients randomized and with available outcome data
- gestational age and weight
- gender
- breast milk or formula or both

**Primary and Secondary Outcomes**

- outcome definition
- time points outcome was collected
- unit of measurement, measure of error (if continuous)

**Appendix C: Methodological issues in previous reviews**

AlFaleh 2013 Cochrane review^11^

- Use of fixed effects versus random effects model of data synthesis.
- Did not follow intention to treat - did not count all randomized patients when extracting data.
- Data for outcomes of interest reported in trials as medians were transformed into means.
- Methodological issues found in the reporting of specific trials included in this review are as follows:

Bin-Nun 2005^31^

- Initiation of enteral feeds / start of probiotics was reported in trial, but was not included in the analysis.
- Time to reach full feeds was reported in the trial, but not reported in the analysis.

Braga 2011^32^

- Risk of Bias: low risk of selected reporting, but secondary outcomes listed in trial registration are not reported (e.g. time to full enteral feeds, duration of hospital).
- Duration of therapy not clearly defined in study, but was included in the analysis.
- It was not clear whether total sepsis or culture-proven sepsis was reported by the study.
- ITT results on mortality were not reported.

Costalos 2003^33^

- Risk of Bias:
  - Did not assess selective reporting
  - Study reported allocation was concealed in envelopes. This was assessed as low risk despite being unclear whether the envelopes were opaque.

Dani 2002^34^

- NEC-related death was analyzed as overall mortality.

Demirel 2013^35^

- Time of initiation of therapy was reported in the trial, but was not included in the analysis.

Fernandez-Carrocera 2013^36^

- Categorization of outcomes into subgroups based on duration was not clearly stated in the trial.
- Culture-proven sepsis was reported in trial, but was not included in the analysis.

Kitajima 1997^37^

- Unclear how mortality and sepsis number counts were obtained (not reported in original publication).

Li 2004^38^

- Included in review, but does not contribute to any outcomes because of lack of data (e.g. incidence of NEC). Reporting of this decision was not explicit.

Lin 2008^40^

- Withdrawals after randomization were not included in the final analysis.

Millar 1993^44^

- Classified under wrong species subgroup (analysis 4.2). Intervention was single species Lactobacillus but was analyzed in multi-species subgroup.

Manzoni 2006^41^

- NICU length of stay was reported in the trial, but was not included in the analysis.

Mihatsch 2010^43^

- Definition of sepsis was not clear. Paper reports number of positive blood cultures. Unclear whether this was number of cultures or number of patients with positive culture. This was recorded as number of patients in the anlysis.
- Time to full feeds was reported in the <1500g subgroup, but was not included in the analysis.

ProPrems 2013^46^

- For the <1000g subgroup:
  - Culture-proven sepsis was analyzed using overall study population data.
  - Mortality was analyzed using overall study population data.

Reumann 1986^47^

- Mortality data was available for subgroup by species but was not included in the analysis.

Rojas 2012^48^

- Denominators used in the <1500g subgroup were those of the overall study population.

Romeo 2011^20^

- Sepsis outcomes were reported in the trial, but were not included in the analysis/
- Events in the control group were not divided between the two active treatment arms resulting in double counting.

Sari 2011^51^

- All-cause mortality was reported in the trial, but was not included in the analysis.

Stratiki 2007^52^

- Incorrect denominators used for analysis.

Yang 2014 systematic review^12^:

- Included trials of stage 1 NEC or above - unusual inclusion for this type of review. Use of random effects to synthesize data only after statistical testing for heterogeneity, rather than deciding a priori based on trial characteristics
- Methodological issues found in the reporting of specific trials included in this review are as follows:

Fu 2012^62^

- Was not a randomized trial.

Huang 2009^14^

- Was not a randomized trial.

Ke 2008^13^

- Did not use Bell’s for criteria for diagnosis of NEC.

Li 2011^64^

- Was not a randomized trial.

Yang 2011^19^

- Definition of NEC was unclear (Stage 1 included?).

Hunter 2012^63^

- Not a randomized trial (although it did not contribute to any of the outcomes in the graphs).

Supplemental Figure 1: Study quality and risk for bias assessment for included studies


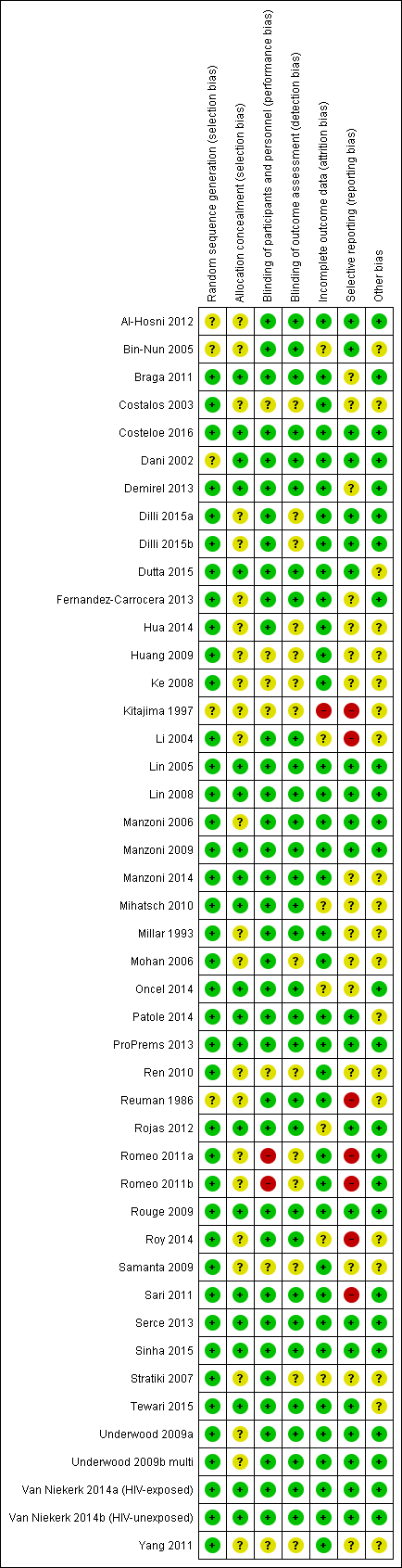


Supplemental Figure 2: Forest plot showing the effect of probiotics on severe NEC subgrouped by timing of initiation of therapy


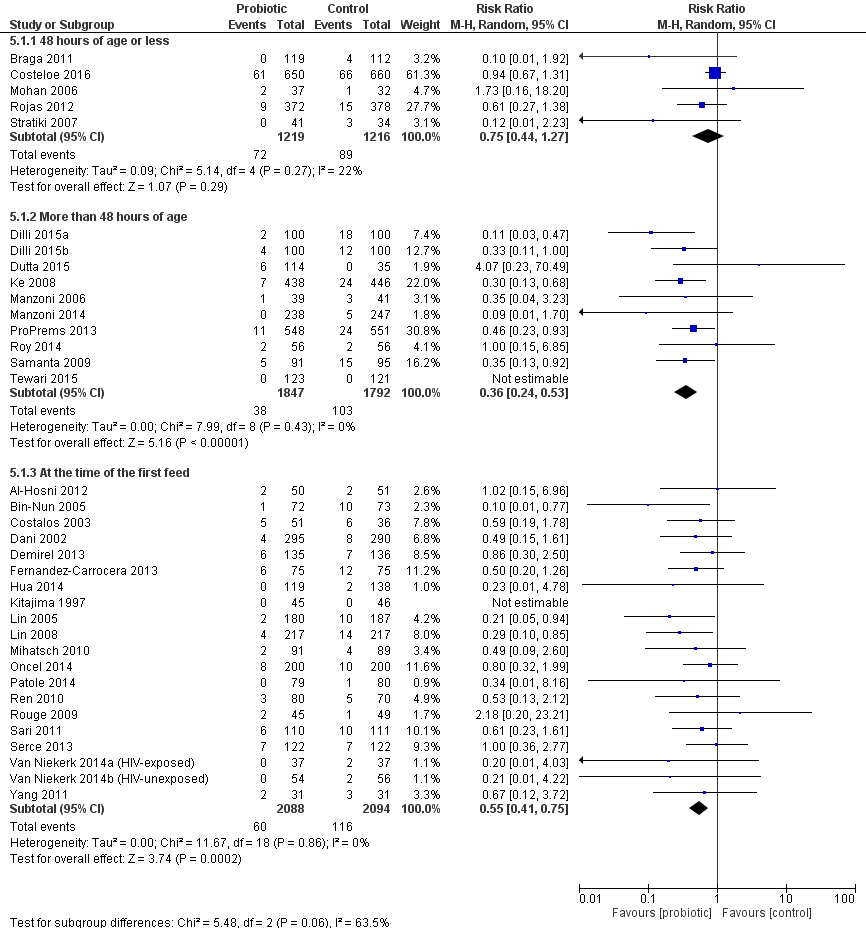


Supplemental Figure 3 - Forest plot showing the effect of probiotics on severe NEC subgrouped by duration of therapy


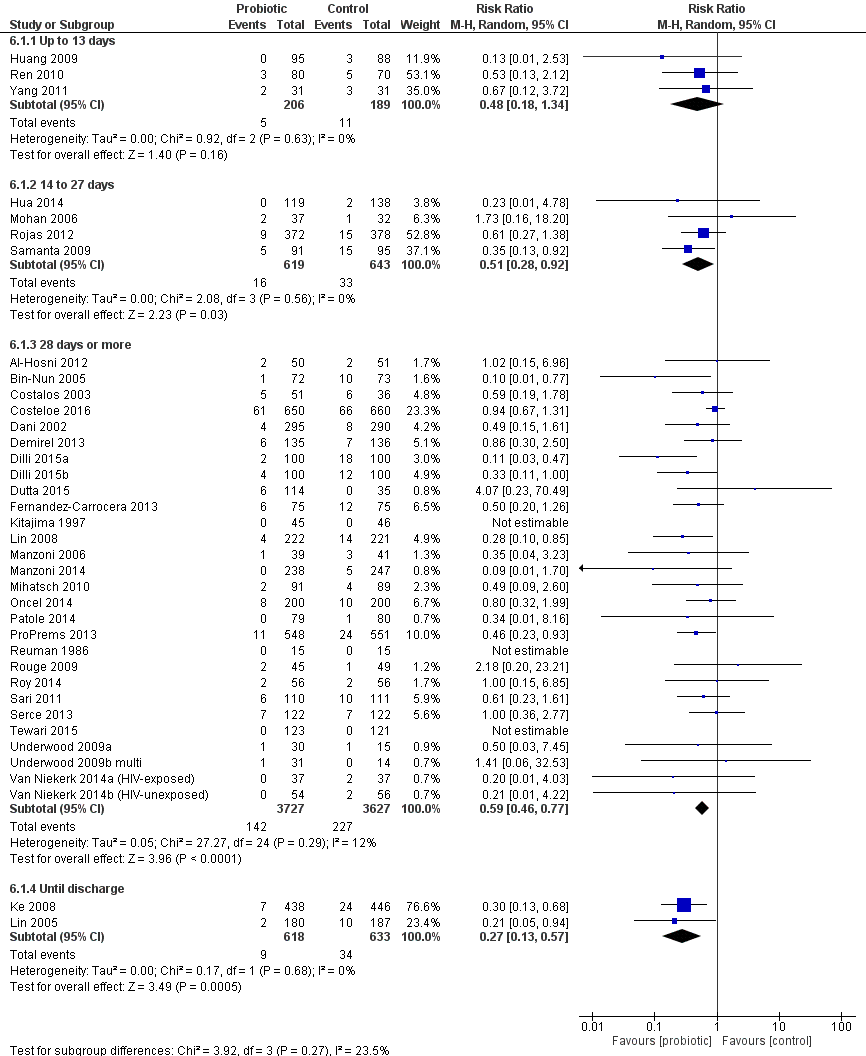


Supplemental Figure 4: Forest plot showing the effect of probiotics on NEC subgrouped by species


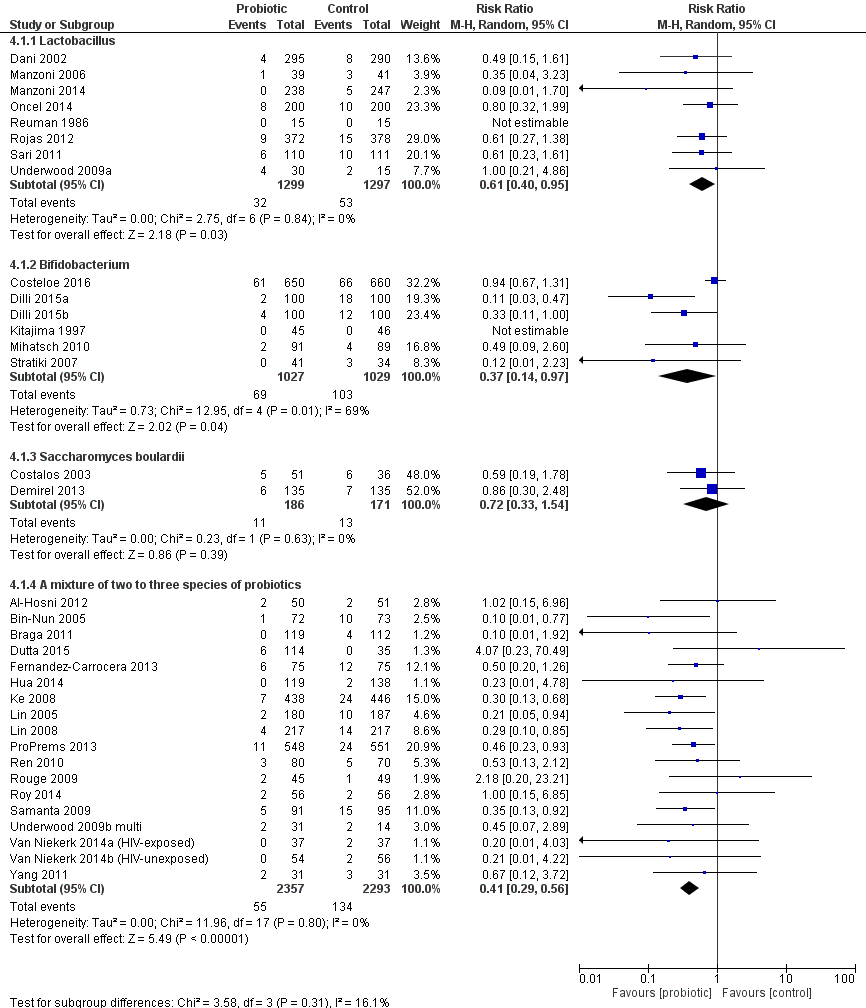

Supplement: Supplemental Information 2 [file peerj-04-2429-s003.docx]
